# Animal organoids as transformative platforms for viral infections and zoonotic cross-species viral research

**DOI:** 10.1128/jvi.02197-25

**Published:** 2026-03-03

**Authors:** Inés García-Rodríguez, Isabel García-Dorival, Covadonga Alonso, Miguel Ángel Cuesta-Geijo

**Affiliations:** 1 Departamento de Biotecnología, CSIC-INIA, Instituto Nacional de Investigación y Tecnología Agraria y Alimentaria 54402, Madrid, Spain; 2 National Center of Microbiology (CNM), Instituto de Salud Carlos III (ISCIII) 38176, Madrid, Spain; Indiana University Bloomington, Bloomington, Indiana, USA

**Keywords:** organoids, virus-host interactions, zoonotic infections, veterinary research

## Abstract

Organoids have revolutionized human biomedical research since their development in 2010. However, as nearly 75% of human infectious diseases originate in animals, animal-derived organoids are essential to complement human models, yet their development in veterinary contexts remains scarce. Organoids replicate native tissue architecture, enabling species-specific and comparative studies of viral infection and host response. Thus, animal organoids represent a powerful field in organoid technology, advancing cross-species virology research and strengthening strategies for zoonotic disease preparedness.

## INTRODUCTION

As obligate intracellular parasites that rely on host cellular machinery for replication, viruses recall appropriate experimental models for elucidating virus-host interactions and pathogenic mechanisms. Traditionally, virology research has relied on two principal approaches: two-dimensional (2D) cell monolayers and *in vivo* animal models. Each methodology has contributed substantially to fundamental understanding of viral biology and pathogenesis, while presenting significant methodological constraints that would limit their translational relevance.

2D cell cultures provide a standardized and cost-effective tool for studies about viral pathogenesis and antiviral drug screening; however, this methodology fails to mimic the complexity and physiological relevance of native tissues (1). As a result, they often lead to limited translational accuracy (2). Animal models offer valuable *in vivo* insights but pose ethical, financial, and practical challenges (3). Importantly, many viruses exhibit strict species specificity, infecting only certain hosts, which limits the availability of adequate *in vivo* models (4). These limitations further highlight the need for alternative methodologies in virology research.

Organoids emerged as *in vitro* promising candidates to bridge this gap. Organoids are three-dimensional (3D) self-organized cells that closely recapitulate the architecture and function of the organ they mimic (5). Unlike conventional cell lines, organoids contain multiple cell types and preserve critical cell-cell interactions in a spatially relevant context (6). Additionally, they can be maintained long-term and are compatible with cutting-edge molecular techniques such as genetic editing, live-cell imaging, transcriptomic or proteomic profiling, or epigenetic analyses enabling mechanistic studies at higher throughput than animal models.

Over the past decade, organoids have emerged as powerful tools in biomedical research, with human-derived models leading the way since the first long-term, self-renewing cultures were established by Hans Clevers and colleagues (7, 8). These advances have accelerated our understanding of human physiology and disease, particularly in areas such as drug screening, personalized medicine, and disease modeling (9, 10).

The usefulness of organoids in virology has been demonstrated through numerous studies in human systems (11). These models have enabled the cultivation of human viruses that were difficult to cultivate, such as norovirus and enterovirus C (12, 13), allowed the modeling of disease phenotypes that accurately reflect clinical pathology (14, 15), and facilitated the selection of physiologically relevant antiviral drugs (16, 17).

However, approximately 75% of human infectious diseases originate from animals (18), highlighting a critical need for organoid systems that can model disease processes in animal hosts with zoonotic potential. In this context, animal organoids do not merely replicate human advances. Zoonotic pathogens predominantly originate in animal reservoirs, and the mechanisms governing cross-species transmission are shaped by host-specific biological determinants that cannot be adequately recapitulated using human-derived experimental systems alone (19, 20). Consequently, organoid models derived from natural or intermediate animal hosts represent an essential platform for dissecting host-pathogen interactions at the species level, particularly during early stages of viral emergence and adaptation, capabilities that human organoids are unable to provide. In addition, viruses exhibit a high degree of species specificity, driven by differences in cellular receptor availability, intracellular replication environments, and host innate immune responses across species, which critically influence viral tropism, replication efficiency, and pathogenesis (21, 22). These host-dependent factors substantially limit the translational relevance of human organoids for studying infections in animal hosts, whereas animal-derived organoids preserve the physiological, molecular, and immunological context required to model viral replication and host responses with higher biological fidelity (8, 23).

Consequently, the development of animal organoids marks a paradigm shift in veterinary and comparative virology, providing physiologically relevant models to investigate viral pathogenesis and zoonotic virus dynamics across species. The integration of human and animal organoid technologies establishes a synergistic framework that advances mechanistic understanding, strengthens early-warning systems for zoonotic threats, and promotes One Health strategies by bridging human, animal, and environmental health.

Within this context, developing robust expertise in the generation and maintenance of organoid models in the laboratory is imperative to fully exploit their translational and predictive potential in infection research and preparedness, while minimizing the need for *in vivo* animal experimentation in line with the principles of reduction and replacement in animal research.

In this review, we provide an overview of the key steps involved in the generation of animal organoids, emphasize their complementarity with human organoids, and explore their current and potential applications in virology.

## ORGANOID DEVELOPMENT

Methodologies established for human-derived organoids can be readily adapted to animal systems. Nevertheless, the widespread adaptation of human organoid protocols to animal systems introduces challenges for cross-species comparability, which are discussed in Box 1. Moreover, ethical and regulatory frameworks differ: while human-derived organoids are subject to stringent biospecimen and ethical governance, animal organoids are regulated under species-specific welfare standards. These regulations are generally less complex than those applied to human research, offering a significant advantage for their development, scalability, and experimental application.

Box 1. **Challenges in standardization of animal organoids for cross-species comparisons**

A major challenge in the application of animal organoids to comparative virology lies in the **lack of standardized, species-tailored protocols**. To date, most animal organoid systems have been established by adapting protocols originally developed for human tissues, including the use of similar growth factor cocktails, extracellular matrices, and differentiation cues. While this approach has accelerated the adoption of organoid technology across species, it also introduces important sources of variability that complicate cross-species comparisons.

First, **species-specific differences in growth factor requirements and signaling pathways** can influence organoid establishment, differentiation efficiency, and cellular composition. Media formulations optimized for human may not elicit equivalent responses in animal cells, potentially resulting in altered differentiation states or biased cell populations. Consequently, organoids from different species cultured under “humanized” conditions may not be developmentally or functionally equivalent.

Second, **differences in differentiation state and maturation** represent a critical confounder. Organoids derived from different species may exhibit varying degrees of differentiation under identical culture conditions, which is particularly relevant for viral tropism and replication studies. Apparent species-specific differences in viral susceptibility may therefore reflect differences in cellular maturity rather than true host restriction.

Third, **variation in receptor expression and entry factor availability** poses a major challenge for comparative virology. Expression levels, isoforms, or spatial localization of viral receptors or co-receptors can differ substantially across species and may be modulated by culture conditions. Without careful characterization, discrepancies in viral infection efficiency across organoids may be driven by culture-induced differences rather than intrinsic host biology.

Addressing these challenges will require systematic **species-specific optimization of culture conditions**, improved benchmarking of differentiation and maturation states, and routine molecular profiling of organoids used for cross-species studies.

The first step to develop animal organoids involves the decision of the starting material. Organoids can be derived from adult stem cells (ASCs), embryonic stem cells (ESCs), or induced pluripotent stem cells (iPSCs) (24). ASC-derived organoids are grown using media enriched with tissue-specific growth factors that maintain adult tissue characteristics and are widely used (5, 7). On the other hand, ESC/iPSC-derived organoids are developed in a step-wise process that mimics the developmental signals involved in organogenesis (5, 25). These models are typically bioengineered, bioprinted, or cultivated in an extracellular matrix (ECM) to promote proper cellular polarization and organization (26, 27).

The generation of organoids from ASCs requires access to tissue samples, but the resulting organoids usually retain adult characteristics, which makes them more representative of the tissue of origin (28). One limitation is that these are restricted to the lineage of the adult organ, but they more faithfully preserve mature cellular phenotypes, tissue-specific architecture, and functional responses of the native tissue (8). Importantly, for viral infection studies, ASC-derived animal organoids provide higher translational relevance by retaining host-specific receptor expression, intracellular replication environments, innate immune responses characteristic of adult target tissues, and higher genetic and epigenetic stability over prolonged culture (9, 29). This process typically involves enzymatic tissue dissociation to isolate the resident stem cells (30); embedment in a matrix, often Matrigel, to facilitate 3D organization (10); and culture in media containing specific growth factors to promote differentiation to a specific organ.

For ESC/iPSC-derived organoids, the main advantage is that a range of different organoids can be generated from a single cell source, offering superior scalability and developmental versatility than ASC-derived organoids. These recapitulate early embryonic programs and could generate multiple lineages across germ layers, enabling the study of organogenesis and congenital disease processes (31). However, iPSC-derived organoids usually maintain a fetal-like phenotype which can limit their relevance for modeling infections that preferentially target differentiated adult cells (28). Moreover, even though iPSCs offer unlimited expansion potential, they may exhibit genetic and epigenetic instability that arises from reprogramming and prolonged culture, affecting experimental reproducibility (32). iPSCs have been developed from several animal species, including pigs, cows, horses, dogs, or chickens (33). However, current applications of this type of animal organoids to date have been limited to date to the development of cerebral organoids from the endangered rhinoceros *Dicerorhinus sumatrensis* (34) and the generation of retinal organoids from rhesus macaques (35).

Together, these distinctions underscore the complementary roles of PSC- and ASC-derived organoids and provide a framework for selecting appropriate model systems according to the biological question addressed. While PSC-derived organoids excel in developmental modeling and scalability, ASC-derived organoids provide superior physiological relevance for viral pathogenesis studies in adult tissues and natural host species.

Since the majority of currently available animal organoids are derived from ASCs (36, 37) and largely represent epithelial tissues, which are key tissue portals for viral entry and replication, we will focus on ASC-derived epithelial organoids and their applications in virology research.

## TECHNICAL APPROACHES FOR ANIMAL ORGANOID INFECTION MODELS

Most progress in this field has been made using mice (7, 25), which have traditionally served as primary models for the development of human organoids. However, recent efforts have expanded the scope of animal organoid technology to include various species of agricultural and biomedical interest. These include farm animals such as pig, goat, sheep, cow, rabbit, or chicken, and also companion animals such as dog or cat (38).

Organoids can be utilized in several complementary ways to analyze the impact of viral infection due to the handful of biological methodologies that could be applied in this setting. Conventional techniques such as viral titration assays and quantitative PCR could be a first approach to quantify viral replication and monitor infection kinetics over time. Structurally, organoids can be imaged post-infection using techniques such as confocal or fluorescence microscopy as useful tools to analyze the biology of viral infection at the cellular level. These methods allow visualization of viral spread and colocalization with specific cellular markers, enabling the characterization of the viral tropism (39). Furthermore, electron microscopy allows a higher resolution analysis and can also be employed to observe virus-induced subcellular changes in organoids, including alterations of subcellular organelles and cellular architecture (40, 41).

To investigate host responses to infection, various analytical approaches can be applied. For example, cytokine profiling of culture supernatants offers a focused view of the virus-elicited innate immune response by quantifying selected proinflammatory cytokines and antiviral mediators (42). To gain a broader and more integrated understanding of the effects of infection on the host, omics approaches can be used such as bulk RNA sequencing or proteomics. These methodologies enable the analysis of global transcriptional or proteomic shifts across the entire organoid, capturing determined host tissue alterations caused by viral infection, including changes in immune signaling, metabolism, or cellular stress responses (14, 43). Additionally, single-cell RNA sequencing can be applied to resolve how distinct cell types within the organoid respond to infection, distinguishing between infected and bystander cells and capturing cell-specific responses (44, 45). Importantly, organoids also support the evaluation of novel clinical interventions, including prophylactic and therapeutic drugs targeting viral genes of host pathways essential for efficient viral replication (46). Importantly, many of these approaches rely on the complexity and cellular diversity that organoids provide, which is absent in traditional 2D cell lines.

## BIOLOGICAL MECHANISTIC INSIGHTS FROM ANIMAL ORGANOIDS IN VIROLOGY

A growing number of animal organoids of several origins have been developed and applied to study viral infections across various species and organ systems, as summarized in Table 1. Among these, porcine organoids are the most extensively used, highlighting swine as a major livestock species and a relevant model for zoonotic diseases (47). Organoids have provided valuable insights into virus-host interactions, cell tropism, innate immune responses, and tissue-specific pathogenesis.

**TABLE 1:** Overview of nonrodent animal-derived organoid models that have been used for viral infection studies, categorized by target organ and virus

Animal	Organoid model	Virus	Reference(s)
Pig	Intestine	Porcine epidemic diarrhea virus (PEDV)	(48–50)
		Porcine deltacoronavirus (PDCoV)	(51, 52)
		Transmissible gastroenteritis virus (TGEV)	(50, 53)
		Senecavirus A (SVA)	(54)
	Airway	Porcine respiratory coronavirus (PRCoV)	(55, 56)
		Swine influenza A virus (IAVsw)	(56–59)
		Porcine hemagglutinating encephalomyelitis virus (PHEV)	(60)
Cow	Intestine	Group A rotavirus (RVA)	(61)
	Airway	Highly pathogenic avian influenza virus (HPAIV)	(62)
		Bovine herpesvirus 1 (BHV-1)	(63, 64)
		Bovine respiratory syncytial virus (BRSV)	(64)
		Bovine parainfluenza virus type 3 (BPIV3)	(64)
Rabbit	Intestine	Rabbit calicivirus Australia-1 (RCV-A1)	(65)
	Liver	Rabbit hemorrhagic disease virus (RHDV)	(66, 67)
Chicken	Intestine	Low pathogenic avian influenza virus (LPAIV)	(68)
Cat	Intestine	Feline coronavirus (FCoV)	(69)
Goat	Mammary gland	Caprine arthritis encephalitis virus (CAEV)	(70)
Bat	Intestine	Severe acute respiratory syndrome coronavirus 2 (SARS-CoV-2)	(71–75)
		Pteropine orthroreovirus (PRV)	(71)
		Marburg virus (MARV)	(76)
		Influenza A virus (IAV)	(74)
		Mammalian orthoreovirus (MRV)	(74)
		Coronavirus HKU4 (CoV-HKU4)	(75)
		Enterovirus A71 (EV-A71)	(75)
	Airway	Marburg virus (MARV)	(76)
		SARS-CoV-2	(74, 77)
		Middle East respiratory syndrome coronavirus (MERS-CoV)	(74)
		IAV	(74)
		Mammalian orthoreovirus (MRV)	(74)
		Pteropine orthoreovirus (PRV)	(77, 78)
	Kidney	IAV	(74)
		Ortho-hantavirus seoulense (SEOV)	(74)
		MRV	(74)
Camel	Airway	SARS-CoV-2	(79)
		MERS-CoV	(79, 80)

Porcine intestinal organoids have been used to study infections by porcine enteric viruses, particularly porcine epidemic diarrhea virus (PEDV). Studies using these models have demonstrated that different types of interferon (IFN) exert distinct antiviral effects on PEDV, as IFN-λ3 had a more potent antiviral activity than IFN-α (48). These organoids also enabled the characterization of PEDV cell tropism, revealing the infection of multiple intestinal cell types, including stem cells and goblet cells (49). Similar work using porcine deltacoronavirus (PDCoV) revealed the diverse susceptibility to infection of enteroids derived from different intestinal regions. This specificity correlated with the region-specific distribution of the viral receptor, aminopeptidase N (APN) (52). PDCoV infection of enterocytes was consistent with *in vivo* findings (81). Moreover, a comparison between PDCoV-infected organoids and a significant activation of innate immune gene expression was obtained using enteroids but not in infected cell lines (51). Notably, porcine enteroids sustain long-term cultures (up to 6 months), during which they retain their ability to differentiate, remaining susceptible to multiple porcine enteric viruses such as PEDV and transmissible gastroenteritis virus (TGEV) (50). This enables repeated use in experiments without the need for fresh tissue to generate new organoids.

A recent study demonstrates that Senecavirus A (SVA) efficiently replicates in apical-out porcine intestinal organoids, a physiologically relevant model that exposes the apical surface of the intestinal epithelium to viral infection. SVA induces diarrhea, dehydration, and mortality in piglets. Notably, infection induces the formation of stress granules and activates the innate immune response, revealing host-pathogen interactions that are difficult to capture in traditional 2D cell cultures. By linking viral replication with tissue-specific immune responses in a 3D organoid system, this work provides an innovative platform for studying SVA pathogenesis, tropism, and potential antiviral strategies in the natural porcine host (54).

Together, porcine intestinal organoids provide a physiologically relevant and scalable platform to dissect cell tropism, region-specific susceptibility, and innate immune responses to enteric viruses, while enabling long-term experimentation and significantly reducing reliance on animal infection models.

In the respiratory context, porcine airway organoids have been used to explore tissue-specific susceptibility to coronavirus. For example, porcine respiratory coronavirus (PRCoV) was able to replicate in airway organoids, whereas TGEV could infect both airway and intestinal models, demonstrating the importance of organ-specific tropism (55). Organoid-derived airway monolayers have been used to study swine influenza A virus (IAVsw) and correlate well with severity of infection of the corresponding strain. Infection with the IAV H3N2 strain, which causes severe disease in pigs, led to a greater disruption of epithelial integrity in airway epithelium cultures, as measured by reduction in trans-epithelial resistance (TEER) and greater damage to tight junctions. In contrast, H1N2 or H1N1, strains that are associated with mild disease in pigs, caused less disruption to epithelial integrity, showing that this model is useful for assessing virulence of viral strains (57). In line with these findings, other studies showed that H1N1 did not reduce TEER because, despite the loss of cilia in the apical surface, basal cells were able to repopulate the monolayer (59). Finally, airway monolayers have been used to study porcine hemagglutinating encephalomyelitis virus (PHEV) infection. This study revealed early innate immune activation and downregulation of cilia-associated genes (60), findings that are consistent with *in vivo* observations of mucociliary loss (82).

Beyond pigs, bovine organoids have been developed for virology studies. Bovine enteroids, together with human-derived enteroids, have been used in investigations of host-specific mechanisms in rotavirus infection, driving to the discovery of specific interactions with viral receptors that are responsible for species-specific infection of the host (61). Tracheal organoids were used to model bovine herpesvirus 1 (BHV-1) infection and testing an immunomodulator. This system was useful to show that this treatment was insufficient to reduce infection (63). Airway monolayer cultures have been applied to compare infection of different bovine respiratory viruses, including bovine respiratory syncytial virus (BRSV), BHV-1, and bovine parainfluenza virus type 3 (BPIV3), showing different interactions with the airway epithelium (64).

Rabbit liver organoids have emerged as a powerful *in vitro* model for studying rabbit hemorrhagic disease virus (RHDV). When differentiated into monolayers, these 3D cultures demonstrated robust replication of multiple RHDV genotypes, a capability absent in analogous monolayers derived from cat and mouse liver, a finding that underscores the virus’s strict species specificity (66). Remarkably, serial passaging of RHDV in this system was enabled by inhibition of IFN response revealing a critical role for innate immune evasion (67). In contrast, intestinal rabbit organoids failed to support infection of the enterotropic rabbit calicivirus Australia-1 (RCV-A1), likely due to the absence of specific cellular targets required for viral entry or replication within the engineered tissues (65). These contrasting outcomes highlight the utility of organoid models for dissecting viral tropism mechanisms, including receptor specificity and host-pathogen interactions, while emphasizing the importance of tissue- and species-specific cellular composition in infection studies.

Chicken intestinal organoids have enabled the study of low pathogenic avian influenza virus (LPAIV). Infection with the H6N1 strain triggered a strong interferon-stimulated gene response, while H9N2 did not induce such a significant innate immune activation, despite both strains achieving similar replication levels (68). These results suggest that viral pathogenesis may not always correlate with replication efficiency, but rather with differential host responses.

Organoid models have emerged as valuable tools for studying veterinary and zoonotic pathogens in less commonly studied species. Feline enteroids, for instance, have enabled groundbreaking studies of feline coronaviruses (FCoVs), including the first successful *in vitro* replication of mild feline enteric coronavirus (FECV), unachievable in traditional 2D feline cell lines due to the virus’s strict dependence on intestinal epithelial differentiation states (69). Caprine mammosphere models (3D mammary epithelial structures derived from goat tissue) have provided insights into caprine arthritis encephalitis virus (CAEV) pathogenesis, with infection inducing severe disorganization of the mammary organoids, including loss of polarity and luminal collapse, thereby modeling the tissue-destructive effects observed *in vivo.* (70).

Together, these studies demonstrate that animal organoids are indispensable tools for virological research, as they recapitulate species- and tissue-specific viral tropism, replication, and host responses that cannot be accurately modeled in conventional cell lines or human organoid systems. Beyond studying infections by single pathogens, organoids serve as powerful tools to investigate the complex dynamics of co-infections involving multiple pathogens, which is a common occurrence in both human and animal diseases. Organoids allow for a precise control over the order, timing, and combination of the infections, offering insights into the interactions between pathogens and with the host (83). Within animal organoids, a notable example is the use of a porcine airway monolayer model to analyze the interaction of IAVsw and PRCoV. This study demonstrated that prior infection with IAVsw significantly inhibited subsequent PRCoV replication when the interval between infections was 3 days. However, when the interval between infections was extended, significant PRCoV replication was observed, because IAVsw infection increased the expression of APN, the viral receptor for PRCoV. Interestingly, the reverse sequence, PRCoV infection prior to IAVsw, did not impact IAVsw replication (56).

## ZOONOTIC INFECTIONS

**Fig 1:**
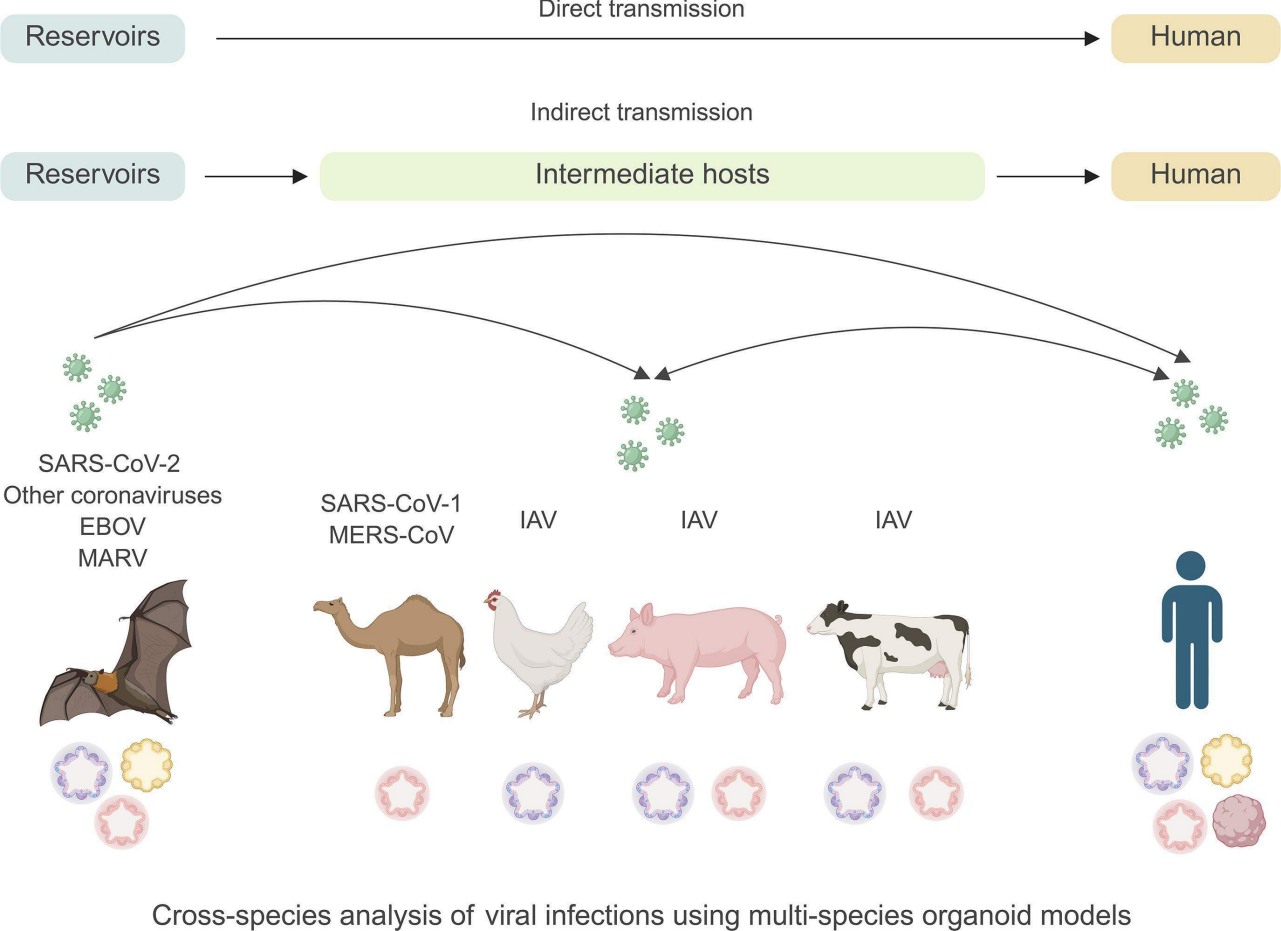
Schematic representation of the use of organoids from different animal species to study the effects of viral infection across hosts. This figure was created with BioRender.

Infectious diseases pose a risk for zoonotic spread, which means that infections are spread between animals and humans (84). It has been estimated that 60% of emerging human infectious diseases are of zoonotic origin (84); thus, the study of the infection in their primary or intermediate hosts is key to understanding disease spillover (Fig. 1).

Bats constitute a large reservoir population of zoonotic viruses, including Middle East respiratory syndrome coronavirus (MERS-CoV), SARS-CoVs, rabies virus, Nipah virus, Marburg virus (MARV), or Ebola virus (77). Organoid models have become valuable tools in exploring virus-host interactions across bat species and several bat tissues. For instance, enteroids derived from horseshoe bats (*Rhinolophus sinicus*) have demonstrated the capacity to support replication of several human isolates of SARS-CoV-2, suggesting that bat intestinal cells could potentially support infection by SARS-CoV-2 or related progenitor viruses (72). Another study using a similar model also showed replication of SARS-CoV-2 and a bat coronavirus HKU4 (CoV-HKU4). Interestingly, they were resistant to infection by enterovirus A71 (EV-A71), a virus exclusive to humans, indicating that these models faithfully reproduce the natural susceptibility (and resistance) patterns seen in bats, highlighting their biological relevance (75).

In contrast, airway organoids from a different bat species (*Eonycteris spelaea*) did not support the replication of SARS-CoV-2 but were permissive to pteropine orthoreovirus (PRV3M), for which these bats are considered the main reservoir host (77). Similarly, enteroids from *Rousettus leschenaultia* bats also failed to support SARS-CoV-2 replication (71). Further studies using respiratory organoids from multiple bat species discovered that only Rhinolophus bats were susceptible to SARS-CoV-2 infection (74). Furthermore, airway and intestinal organoids derived from *Rousettus aegyptiacus* have shown to support MARV replication. Interestingly, these organoids trigger a robust innate immune response upon infection, as shown by RNA-seq, a feature notably absent in comparable human organoids (76). Collectively, these findings indicate that the development of bat organoids from different species and organs can be a potent tool to establish zoonotic infection origin and further understand virus-host interactions and pathogenesis of natural reservoir hosts (85). Beyond mechanistic studies, bat organoids have also proven successful isolation of previously unknown viruses by isolation of bat fecal samples, allowing for surveillance and enhanced pandemic preparedness (74). By enabling controlled infection studies in an otherwise experimentally inaccessible animal, bat organoids constitute essential platforms for understanding zoonotic virus origin, host specificity, and spillover risk. Birds are natural reservoirs for IAV that has adapted to humans, causing several pandemics throughout history (86, 87). Infection using both human and chicken distal airway organoids has established the susceptibility of each host to various IAV strains, and direct comparison between these models can yield valuable insights into the mechanisms of cross-species transmission and adaptation (88). More recently, IAV infections have also been reported in bats (89), prompting the use of bat airway organoids that have shown susceptibility to several IAV strains of human, avian, and bat origin (74). Additionally, bovine airway monolayer cultures were used to assess the infection of highly pathogenic avian influenza (HPAI). These models demonstrated that multiple lineages of H5N1 influenza can infect the respiratory tract, highlighting the importance of avian influenza surveillance in cattle (62).

Finally, it is also important to address the presence of additional hosts in the spreading of zoonotic diseases. For instance, although MERS-CoV and SARS-CoV-1 were likely derived from strains found in bats, palm civets and dromedary camels are believed to be the hosts that introduced them into the human population. Studies using airway models derived from camels have demonstrated their susceptibility to MERS-CoV, but not to SARS-CoV-2, suggesting they do not play a role in the spread of the latter (79, 80). Developing animal organoids of the different species implicated (or not) in the dissemination of the virus could be developed to design a strategic plan to tackle a specific disease by understanding the susceptibility of possible hosts in the field, helping to determine its evolution and pathogenesis before affecting humans.

## DISCUSSION AND FUTURE PERSPECTIVES

There has been considerable debate regarding human organoids, their generation, and their potential applications. Nevertheless, to investigate zoonotic viral infections and cross-species transmission, it is equally essential to develop and utilize animal organoids. Beyond their relevance for human infectious disease research, animal organoids hold substantial value for animal health, wildlife disease ecology, and surveillance of natural viral reservoirs.

In this review, we describe the growing potential of animal organoids as innovative tools to study viral infections and their critical role to advance our understanding of zoonotic diseases. By providing a physiologically relevant platform, organoids enable detailed study of virus-host interactions. Because organoids can be derived from a variety of species, they offer a unique opportunity to model viral infections not only in natural hosts but also in potential intermediate hosts. These comparative models are invaluable to identify novel viral reservoirs and elucidate host-specific factors that shape infection outcomes. For instance, the unique antiviral tolerance of bats compared to humans may underlie their role as major viral reservoirs (90). Comparative organoid models can therefore help elucidate how host response influences viral persistence, pathogenesis, and transmission dynamics, as well as how cross-species infection drives viral mutation and adaptation that could render new hosts susceptible to infection. Such insights are essential not only for anticipating zoonotic spillover but also for improving veterinary disease control and wildlife conservation.

The use of animal organoids aligns with the 3Rs concept: replacement, reduction, and refinement in research (91). Organoids can replicate hallmarks of viral infection, reducing reliance on live animals for veterinary virology and antiviral drug screening. Because multiple organoids can be generated from a single animal, they enable parallel studies across tissues, maximizing experimental yield. Moreover, the physiological relevance of organoids often surpasses that of conventional 2D models. As such, organoids may present a more accurate *in vitro* model for primary drug screening and pathogenesis studies, potentially improving the low translational success of preclinical research (2) while decreasing the number of animals needed at later stages of drug development.

Despite significant advancement in the organoid field, several challenges still persist. While ASC-derived organoids are able to replicate the epithelium of an organ, they lack the mesenchymal component that modulates epithelial responses *in vivo*. Conversely, ESC and iPSC-derived organoids tend to exhibit a more fetal-like phenotype and may not fully recapitulate the functional maturity of adult tissues (28). However, further efforts should be devoted to advancing ESC and iPSC-derived animal organoids, as these systems enable the generation of multiple organ types and allow modeling of organs that cannot be derived from ASCs, including the brain, liver, heart, or pancreas. Scalability also remains a challenge as organoid generation relies heavily on manual handling, they have size inconsistencies, and they need 3D environments that are not readily applicable for standard automated platforms (92). Reproducibility and replicability between laboratories are still ongoing challenges in the organoid field, so new standards to enhance reproducibility are promoted, like transparency in the reporting, harmonization of protocols (93), and monitoring of phenotypic changes in long-term cultures (28).

Another important consideration to improve organoid relevance and reliability is donor diversity. Organoids can be derived from individuals of different ages (94) and sexes (95), allowing researchers to model how biological variables influence susceptibility to infection and therapeutic response. For instance, human gastric organoids have shown age-dependent susceptibility to SARS-CoV-2 (96). Similarly, pediatric nasal organoids have shown higher susceptibility to RSV as compared to adult nasal organoids (97). Sex-based differences in gene expression have been observed in mouse enteroids (98), which may have implications for infection dynamics across sexes. Incorporating this biological variability into organoid studies is essential to improve the predictive value and translation potential of organoids.

Further innovations in organoid technology are likely to expand their applicability in virology, particularly through the integration of vascular and immune components. A major limitation of conventional organoids is the absence of a functional immune compartment and the complex multicellular microenvironment present *in vivo*, which restricts their ability to recapitulate key immune processes such as immune cell recruitment, antigen presentation, adaptive immune responses, and systemic inflammation (29, 99). While epithelial organoids are a valuable model to model innate epithelial responses, such as cytokine production upon viral infection, the absence of immune cells limits investigations related to immune-mediated pathology and viral immune evasion mechanisms. This limitation is particularly relevant for animal viruses in which immune responses critically influence pathogenesis, such as foot-and-mouth disease virus (100), African swine fever virus (101), IAV (68), and porcine reproductive and respiratory syndrome virus, which could not be fully studied in conventional organoids (102, 103).

To address the lack of immune competence, several strategies have been developed. Co-culture approaches that incorporate innate or adaptive immune cells into organoid systems enable partial reconstruction of epithelial-immune interactions and tissue-resident immunity, allowing more physiologically relevant studies of local immune surveillance and antiviral defense (104, 105). In parallel, tonsil-based culture systems have emerged as powerful platforms for virology research. By using tonsil tissue, these models preserve key features of mature lymphoid architecture and enable the study of adaptive immune responses, including T- and B-cell interactions, germinal center-like activity, antibody class switching, and responses to viral antigens or vaccines. As such, they are particularly valuable for studying species-specific antiviral immunity, immune modulation by viruses, and vaccine evaluation in a physiologically relevant context (106, 107).

However, despite their frequent designation as “organoids,” these systems do not meet the strict definition of organoids. They are not derived from stem or progenitor cells, do not undergo self-organized development, and lack long-term growth and self-renewal. So far, they represent *ex vivo* cultures of mature immune tissue. These limitations should be acknowledged to avoid conceptual ambiguity, while recognizing their strength in applications as functional immune models for virology.

Beyond immune integration, vascularization represents another critical frontier in organoid innovation. The absence of vasculature limits nutrient exchange, tissue maturation, and the study of systemic viral dissemination. Microfluidic and organoid-on-chip systems can introduce vascularization and mechanical cues that enhance tissue differentiation and organization (108). These systems have already been applied to model SARS-CoV-2 and IAV infection in airway chips (109) and hepatitis B virus in liver chips (110). Recent breakthroughs combining vascularization with immune-competent organoid systems hold promise for these platforms to provide a more comprehensive and physiologically relevant modeling of viral pathogenesis.

Looking ahead, several concrete priorities will be critical to maximize the potential of animal organoids in virology. First, the systematic development of immune-competent animal organoid platforms that integrate species-specific innate and adaptive immune components will be essential to model antiviral immunity and immune pathology. Second, organoids from key reservoir, livestock, and companion species should be embedded into viral surveillance pipelines, where species- and tissue-specific organoids can be used to assess host range, tissue tropism, replication fitness, and pathogenic potential of newly detected or re-emerging viruses, thereby informing risk and early intervention strategies (74). Third, there is an urgent need for scalable and standardized organoid formats that are compatible with miniaturization, automation, and high-content readouts, enabling high-throughput screening of emerging viruses and antiviral interventions across diverse animal hosts. In conclusion, animal organoids represent a powerful and ethically responsible approach to advance veterinary virology and zoonotic disease research, for which the study of not only the end host (human) but also the intermediate hosts is crucial. Their capacity to model interspecies infection, virus-host interactions, and individual variability makes them indispensable tools for the research of infectious diseases. Their relevance extends beyond human health, with critical applications for animal health, wildlife disease monitoring, reservoir host characterization, and integrated One Health strategies. Enhancing donor diversity, incorporating vascular and immune components, and developing scalable high-throughput technologies will be a key to maximizing the power of organoids as translational tools in virology.
